# Interleukin Dynamics and Their Correlation with Tumor Aggressiveness in Colorectal Carcinoma

**DOI:** 10.3390/ijms26147027

**Published:** 2025-07-21

**Authors:** Elena-Teodora Tâlvan, Liviuta Budișan, Călin Ilie Mohor, Valentin Grecu, Ioana Berindan-Neagoe, Victor Cristea, George Oprinca, Adrian Cristian

**Affiliations:** 1 Faculty of Medicine, “Lucian Blaga” University of Sibiu, 550169 Sibiu, Romaniaadrian.cristian@ulbsibiu.ro (A.C.); 2 Research Center for Functional Genomic, Biomedicine and Translational Medicine, “Iuliu Hațieganu” University of Medicine and Pharmacy Cluj-Napoca, 400012 Cluj-Napoca, Romania; liviuta.petrisor@umfcluj.ro (L.B.); ioananeagoe29@gmail.com (I.B.-N.); victor_cristea@yahoo.com (V.C.); 3 Faculty of Engineering, “Lucian Blaga” University of Sibiu, 550025 Sibiu, Romania; valentin.grecu@ulbsibiu.ro

**Keywords:** colorectal cancer, interleukins, cancer progression, biomarkers, invasiveness

## Abstract

Colorectal cancer (CRC) is a major global health concern, with tumor progression closely influenced by inflammatory mechanisms and cytokine signaling. This study investigates the serum expression levels of interleukins IL-8, IL-17A, and IL-33 in patients with colon cancer, analyzing their association with tumor grade and depth of invasion. The cohort included 42 patients stratified by tumor differentiation (G1–G3) and various invasion types. ELISA assays revealed that IL-8 levels were highest in well-differentiated tumors and in cases of submucosal and serosal invasion, suggesting a key role in early stage inflammation and angiogenesis. IL-17A and IL-33 levels declined progressively with tumor dedifferentiation and increased invasion depth, indicating immune suppression in advanced stages. Multiple regression analyses highlighted a nonlinear, significant relationship between IL-8 and IL-17A, whereas IL-33 showed no direct correlation with other interleukins. A combined model incorporating IL-8, IL-17A, IL-33, and tumor grade accounted for over 70% of IL-17A variability, underscoring their interactive role in CRC biology. These findings support the potential utility of interleukins as biomarkers and therapeutic targets for stratified CRC management.

## 1. Introduction

Colon cancer is one of the most common malignancies worldwide, with a significant public health burden in both developed and developing regions. Globally, approximately 1.2 million new cases of colon cancer are diagnosed each year, leading to around 600,000 deaths annually [1]. The highest incidence rates are observed in high-income countries, especially in Western regions. In the United States, colon cancer remains one of the leading causes of cancer incidence and mortality, although the age-adjusted incidence has declined by 30% over the past decade in adults aged 50 and older due to widespread colonoscopy screening [2]. However, early onset colon cancer is rising among younger adults, particularly white and black populations [3]. In Europe, incidence varies by country but remains among the highest globally, with rates notably high in countries, such as the United Kingdom and Italy [4]. For example, in England and Wales, about 17,000 new cases are diagnosed annually, with a slight upward trend in men over the past decades [5].

Biomarkers are essential tools for diagnosing, monitoring, and personalizing treatment across a wide range of diseases—from chronic and autoimmune conditions to cancers and oral diseases. They enable early detection of systemic illnesses, such as diabetes and cardiovascular disease, guide targeted cancer therapies and help manage autoimmune disorders [6,7,8,9,10]. Recent research underscores the critical role of inflammation—particularly interleukins—in the development and progression of colorectal cancer (CRC). Chronic inflammatory signaling can create a tumor-promoting microenvironment, with interleukins, such as IL-6, IL-8, IL-10, and IL-17, acting as central mediators [11]. IL-6 promotes CRC cell proliferation and survival via the JAK/STAT3 pathway and is associated with tumor growth and poor prognosis [12]. Genetic variants in the IL-6 promoter (e.g., IL6-174C>G) have been linked to increased CRC risk and influence tumor behavior [13]. IL-10, although anti-inflammatory, has been shown to have dual roles, with certain polymorphisms (e.g., IL-10-592C/A) being protective against CRC in some populations [14]. Moreover, IL-17A polymorphisms have been associated with tumor location and progression, highlighting their potential as both biomarkers and therapeutic targets [15]. Collectively, these findings suggest that interleukins play multifaceted roles in CRC biology, influencing disease risk, prognosis, and therapeutic response.

Interleukin-8 (IL-8), a chemokine from the CXC family, plays a significant role in the progression of colorectal cancer (CRC) by promoting inflammation, angiogenesis, and metastasis. IL-8 is produced by tumor cells and cells in the tumor microenvironment, acting through its receptors CXCR1 and CXCR2 to stimulate cell migration, proliferation, and survival. Overexpression of IL-8 has been correlated with poor prognosis, advanced tumor stage, lymphatic and liver metastases, and resistance to chemotherapy [16]. Mechanistically, IL-8 enhances colorectal cancer cell migration via upregulation of integrin αvβ6 through the ERK/Ets-1 signaling pathway [17]. Additionally, IL-8 fosters cancer stem cell maintenance and epithelial–mesenchymal transition, contributing to therapy resistance and tumor heterogeneity [18]. Serum levels of IL-8 have also shown diagnostic and prognostic value in CRC patients, with higher levels associated with worse survival outcomes and disease progression [19]. Collectively, these findings underscore IL-8 as a critical driver in CRC pathogenesis and a promising target for both diagnosis and therapy. Interleukin-17 (IL-17), primarily produced by Th17 cells, is a pro-inflammatory cytokine that plays a complex role in the development of colorectal cancer (CRC). While IL-17 promotes immune responses against pathogens, in the context of cancer, it fosters a chronic inflammatory environment that supports tumor growth, angiogenesis, and immune evasion. Studies have shown that IL-17A and its receptor, IL-17RA, are overexpressed in CRC tissues and are associated with tumor progression and poor prognosis [20,21]. IL-17A has also been implicated in promoting resistance to immunotherapy and chemotherapy, such as cisplatin, by enhancing tumor cell survival and suppressing anti-tumor immunity [22,23]. Interestingly, some studies also suggest a dual role for IL-17, with evidence indicating its association with improved immune activation and better prognosis under specific conditions [24], suggesting that its function may depend on the tumor microenvironment and disease stage. Overall, IL-17 is a promising biomarker and therapeutic target in CRC, reflecting both its immunomodulatory and tumor-promoting roles. Interleukin-33 (IL-33) is a member of the IL-1 cytokine family that plays a dual role in colorectal cancer (CRC), acting as both a promoter and potential suppressor of tumor development depending on the context of the tumor microenvironment [11]. In several experimental and clinical studies, IL-33 has been shown to contribute to CRC progression by promoting tumor growth, angiogenesis, immune suppression, and metastasis. It does so primarily through its receptor ST2, which facilitates the recruitment of tumor-infiltrating regulatory T cells and the remodeling of the tumor microenvironment [25,26]. Elevated serum and tissue levels of IL-33 have been observed in CRC patients and are associated with poorer differentiation and more advanced disease stages, suggesting its potential as a diagnostic biomarker [27]. Conversely, some studies have indicated that IL-33 may stimulate anti-tumor immunity and limit sporadic CRC progression by enhancing interferon-γ responses [28]. This dual nature highlights the complexity of IL-33’s role in CRC and supports ongoing research into IL-33/ST2 as a therapeutic target.

Considering the critical role of biomarkers in understanding the molecular biology of colorectal cancer, in the present study we examined the expression levels of IL-8, IL-17A, and IL-33 in relation to tumor grade and invasion, while also evaluating the correlations between these three interleukins.

## 2. Results

### 2.1. Demographic Analysis

**Figure 1:**
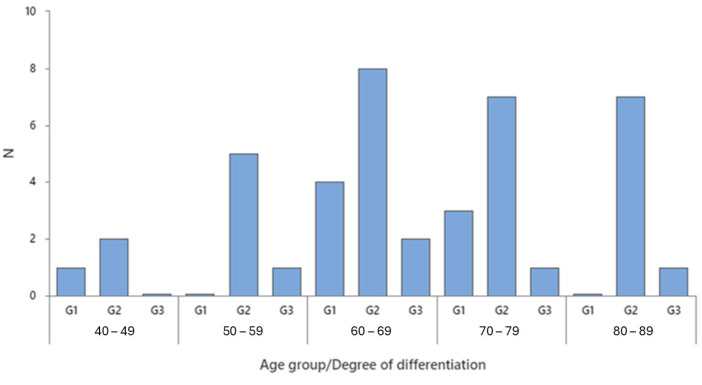
Tumor grading in different age groups.

Figure 1 illustrates the distribution of colon cancer patients across different age groups and degrees of tumor differentiation (G1, G2, G3). The most frequently observed tumor grade across all age groups was G2 (moderate differentiation), with the highest occurrences in the 60–69, 70–79, and 80–89 age brackets, each showing 7–8 cases. G1 (well-differentiated tumors) was less common but showed a consistent presence across age groups, peaking slightly in the 60–69 and 70–79 cohorts. G3 (poorly differentiated tumors) appeared infrequently across all age groups, with the highest count being 2–3 cases. The 60–69 age group exhibited the highest overall number of cases, particularly in the G2 category, indicating a potential age-related peak in moderately differentiated colon cancers. Overall, the data suggest that moderate differentiation (G2) is the most prevalent tumor type, particularly in older age groups.

Table 1 illustrates the mean age distribution of male and female colon cancer patients across three histopathological grades (G1–G3). Among females, mean ages progressively increased with tumor grade: 65.0 years for G1, 67.6 years for G2, and 70.75 years for G3. A similar trend was observed in males, with mean ages of 68.0 years for G1 and 70.93 years for G2; however, only one male patient was recorded in the G3 category, limiting the interpretability for this group. Overall, both genders showed higher mean ages in patients with more poorly differentiated tumors, suggesting a possible association between older age and higher-grade colon cancer. Additionally, standard deviations and interquartile ranges reflected considerable variability within each group, particularly in G2, which also had the largest number of cases for both sexes. These findings may support the role of age as a contributing factor to tumor aggressiveness and underline the importance of stratifying patients by both age and tumor grade in clinical assessments.

Table 2 summarizes the mean age distribution by gender across different types of tumor invasion in colon cancer patients. Overall, male patients tended to be older than female patients across most invasion types. In cases of lymphovascular invasion, males had a higher mean age (71.6 years) compared to females (62.1 years), suggesting a potential age-related vulnerability in men for this invasive pattern. A similar trend was observed in submucosal invasion, with the single male patient being 78 years old versus 56 years in the only female case. For muscular invasion, the limited sample size precludes strong conclusions, though the single male patient was slightly older (67 years) than the female mean (64 years). Organ metastases were more frequent in older individuals overall, with mean ages of 80.0 years in females and 74.0 years in males, indicating that distant spread may occur later in disease progression. In contrast, serous invasion was more common at younger ages for males (mean: 67.6 years) compared to females (mean: 73.3 years), although both groups were generally older than those with earlier-stage invasion. These data suggest a general trend of increasing patient age with more advanced or deeper invasion types and a tendency for males to present with more invasive disease at older ages, particularly in lymphovascular and submucosal involvement. However, interpretation should be cautious given the small sample sizes in some subgroups.

### 2.2. Descriptive Analysis of Interleukin Concentration

Table 3 presents the mean concentrations of interleukins IL-8, IL-17A, and IL-33 across three histological grades of colorectal cancer (G1–G3), illustrating trends in cytokine expression relative to tumor differentiation. The highest mean IL-8 concentration was observed in G1 tumors (39.4 pg/mL), with a wide range and substantial variability (SD = 29.8). In contrast, G2 and G3 tumors showed lower mean values (26.75 pg/mL and 26.73 pg/mL, respectively). An ANOVA was conducted to assess the variation in the mean IL-8 concentration based on the degree of differentiation (G1, G2, G3). The analysis revealed no statistically significant difference in mean IL-8 concentrations across the three tumor grades (F(2, 39) = 0.64, *p* = 0.530). This indicates that while numerical differences in mean concentrations were observed, these differences were not statistically significant at an α = 0.05 level. The G2 group displayed the largest standard deviation (29.85) and an outlier maximum value of 168.56 pg/mL, while G1 also exhibited substantial variability (SD = 29.8). This high standard deviation in both G1 and G2 suggests considerable heterogeneity within these patient subgroups, potentially reflecting diverse underlying biological mechanisms or patient-specific inflammatory responses, which may contribute to the lack of a statistically significant difference despite numerical variations.

**Figure 2:**
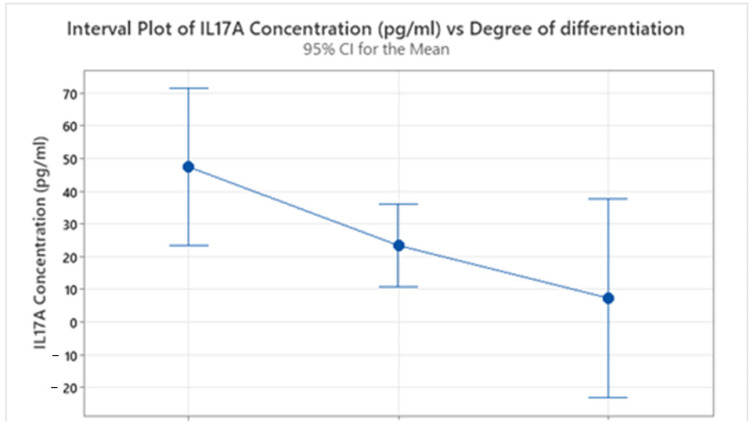
Variation in IL-17A concentration based on the tumor’s degree of differentiation.

IL-17A showed a marked decline with increasing tumor grade. G1 tumors had the highest mean level (47.4 pg/mL, SD = 37.8), followed by a substantial drop in G2 (23.40 pg/mL, SD = 34.64) and a further reduction in G3 (7.306 pg/mL, SD = 1.569), as shown in Figure 2. An ANOVA indicated that the differences in mean IL-17A concentrations across the tumor grades were approaching statistical significance (F(2, 39) = 2.50, *p* = 0.095). While this *p*-value does not meet the conventional α = 0.05 threshold for statistical significance, it suggests a trend of decreasing IL-17A levels with increasing tumor grade. The sharp decline in IL-17A concentrations with poorer differentiation may suggest a diminishing role of Th17-related inflammation in more aggressive tumor phenotypes.

IL-33 levels followed a similar trend, with the highest concentration in G1 tumors (48.1 pg/mL, SD = 57.9), slightly lower in G2 (43.53 pg/mL, SD = 39.10), and notably reduced in G3 (27.3 pg/mL, SD = 29.6). An ANOVA for IL-33 concentrations based on the degree of differentiation revealed no statistically significant difference across the tumor grades (F(2, 39) = 0.40, *p* = 0.672). This indicates that despite numerical variations, the observed differences in mean IL-33 concentrations among G1, G2, and G3 tumors were not statistically significant at an α = 0.05 level. Similar to IL-8, IL-33 concentrations exhibited high variability, particularly in G1 (SD = 57.9) and G2 (SD = 39.10), further indicating heterogeneity in immunological responses within these groups.

The data suggest that all three interleukins—IL-8, IL-17A, and IL-33—tend to be more elevated in well-differentiated tumors (G1) and decline with increasing tumor grade. This pattern may indicate a more active immune or inflammatory response in early stage tumors, which becomes suppressed or altered as the tumor progresses and dedifferentiates. These findings support the potential utility of interleukin profiling as a biomarker strategy for assessing tumor differentiation and immunological activity in colorectal cancer.

The data in Table 4 reflect the distribution of interleukin concentrations (IL-8, IL-17A, and IL-33) across various patterns of tumor infiltration in colon cancer, offering insights into the inflammatory and immunological profiles associated with different stages of tumor invasion. IL-8 levels were highest in cases of submucosal (mean: 36.7 pg/mL) and serous invasion (32.73 pg/mL), with the latter also exhibiting considerable variability (SD: 36.95), likely influenced by extreme values. Lower IL-8 concentrations were observed in lymphovascular invasion (22.57 pg/mL) and organ metastases (24.51 pg/mL), suggesting that IL-8 may be more active during earlier or locally advanced stages of tissue infiltration. IL-17A concentrations showed a contrasting trend, peaking in submucosal invasion (67.88 pg/mL) and serous invasion (34.30 pg/mL), with a marked decrease in organ metastases (7.30 pg/mL) and lymphovascular invasion (13.41 pg/mL). These findings suggest a decline in Th17-mediated immune activity in more advanced disease, particularly in metastatic and lymphovascular contexts. IL-33 concentrations were most elevated in muscular (49.1 pg/mL) and serous invasion (47.2 pg/mL), followed by submucosal (39.26 pg/mL) and lymphovascular infiltration (35.85 pg/mL). Organ metastasis presented the lowest mean IL-33 levels (32.6 pg/mL), reinforcing the notion that IL-33 may play a more prominent role in local invasion and tumor progression rather than in systemic dissemination.

Overall, the interleukin profiles indicate that IL-8 and IL-33 are more closely associated with localized or intermediate stages of tumor invasion, while IL-17A demonstrates a steep decline in advanced disease, potentially reflecting immune suppression during metastatic progression. These cytokines may therefore serve as useful biomarkers for characterizing the invasive behavior and immunological status of colorectal tumors.

### 2.3. Interleukin Correlations in Colon Cancer Grading and Invasion

**Figure 3:**
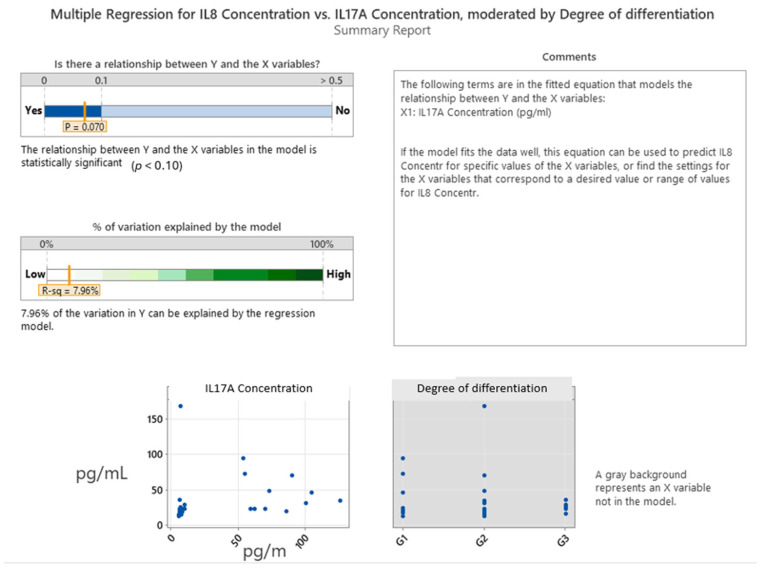
Multiple regression between IL8 and IL17A expression and tumor grade.

The multiple regression analysis in Figure 3 examined the predictive relationship between the IL-8 concentration (dependent variable) and two independent variables: IL-17A concentration and tumor grade (degree of differentiation). This model achieved a *p*-value of 0.070, which is approaching statistical significance at the 0.10 level, indicating a potential—but modest—association between IL-8 and IL-17A levels. The coefficient (0.229) suggests a slight positive linear relationship: as the IL-17A concentration increases, IL-8 levels tend to rise. However, the scatterplot and fitted regression line reveal considerable variability and a weak correlation, supporting only a limited predictive strength. The adjusted R-squared value of 7.96% indicates that less than 8% of the variance in the IL-8 concentration can be explained by the IL-17A concentration alone. The tumor grade (degree of differentiation) did not significantly enhance the model and contributed negligibly to the explained variance, as evidenced by its minimal impact in the incremental R-squared bar chart. Furthermore, the regression of each predictor against the others showed that IL-17A was not collinear with other variables, justifying its inclusion in the model. Still, the weak overall fit highlights that while IL-17A may have a minor role in modulating IL-8 expression, other biological factors likely play a more dominant role in driving IL-8 levels in colorectal cancer. In summary, the analysis suggests a weak but statistically suggestive association between IL-17A and IL-8 concentrations, with no meaningful contribution from tumor grade. This modest correlation points to a limited but potentially biologically relevant link between Th17-related inflammation and IL-8-mediated signaling in the colorectal tumor microenvironment.

**Figure 4:**
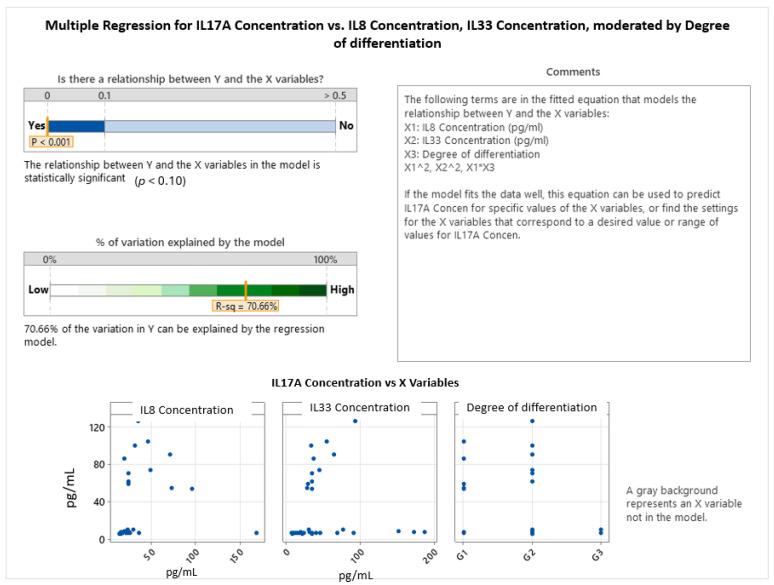
Multiple regression among IL8, IL17A, IL 33, and tumor grading.

The multiple regression model investigating predictors of the IL-17A concentration, incorporating IL-8, IL-33, and tumor grade (degree of differentiation), revealed a statistically significant and robust relationship (*p* < 0.001), with the model explaining 70.66% of the variance in IL-17A levels. This indicates a strong predictive capability when accounting for both cytokine interactions and tumor histological characteristics (see Figure 4).

The final model includes linear, quadratic, and interaction terms, capturing complex, non-linear relationships. The general form of the regression equation for the IL-17A concentration was:

IL17A Concentration = Constant + (Coefficient of X1)·X1 + (Coefficient of X2)·X2 + (Coefficient of X1^2^)·X1^2^ + (Coefficient of X2^2^)·X2^2^

where X1 represents the IL-8 concentration and X2 represents the IL-33 concentration. The specific equations vary based on the degree of differentiation:

- Degree of differentiation: G1

IL17A Concentration = −9.5 + 2.297·IL8 Concentration + 0.744·IL33 Concentration − 0.01954·(IL8 Concentration)^2^ − 0.00455·(IL33 Concentration)^2^

- Degree of differentiation: G2

IL17A Concentration = −58.3 + 3.567·IL8 Concentration + 0.744·IL33 Concentration − 0.01954·(IL8 Concentration)^2^ − 0.00455·(IL33 Concentration)^2^

- Degree of differentiation: G3

IL17A Concentration = −13.9 + 0.83·IL8 Concentration + 0.744·IL33 Concentration − 0.01954·(IL8 Concentration)^2^ − 0.00455·(IL33 Concentration)^2^

**Figure 5:**
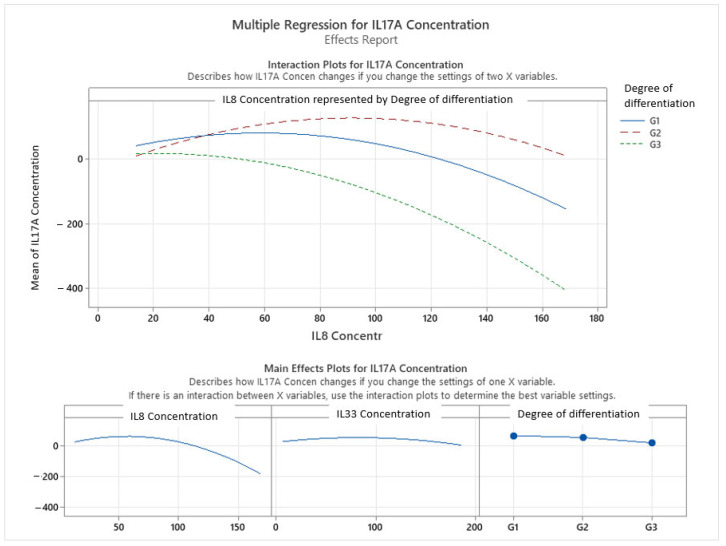
Correlations between the 3 interleukins in colon cancer grading.

Notably, the interaction plot between the IL-8 concentration and tumor grade (Figure 5) vividly illustrates how IL-17A levels vary by both the magnitude of IL-8 and tumor differentiation, exhibiting quadratic correlations. For well-differentiated tumors (G1, represented by the blue solid line), IL-17A expression initially increases with the IL-8 concentration, reaching a peak around 60–80 pg/mL of IL-8, and then begins to decline. This suggests an inverted-U shaped relationship where moderate IL-8 levels are associated with higher IL-17A, but very high IL-8 concentrations might lead to a reduction in IL-17A. A similar, though less pronounced, curvilinear pattern was observed for G2 tumors (red dashed line), where IL-17A levels also increased with IL-8 up to a point before declining, but generally at lower overall IL-17A concentrations compared to G1. In poorly differentiated tumors (G3, green dashed line), IL-17A levels remained consistently lower across the range of IL-8 concentrations and showed a steeper, continuous decline as IL-8 increased, indicating an attenuated or dysregulated inflammatory response in these more aggressive cancers.

The main effects plots (bottom row of Figure 5) further reinforced these curvilinear patterns. The plot for the IL-8 concentration shows a clear inverted-U shape, indicating that IL-17A levels were highest at intermediate IL-8 concentrations and decreased at both very low and very high IL-8 levels. Similarly, the IL-33 concentration also exhibited a curvilinear effect on IL-17A, with IL-17A levels peaking at moderate IL-33 concentrations and declining thereafter. Tumor grade also had a significant influence on IL-17A expression, with higher IL-17A observed in lower-grade tumors (G1) and a marked decline across G2 to G3, as visually represented by the distinct curves for each grade in the interaction plot.

**Figure 6:**
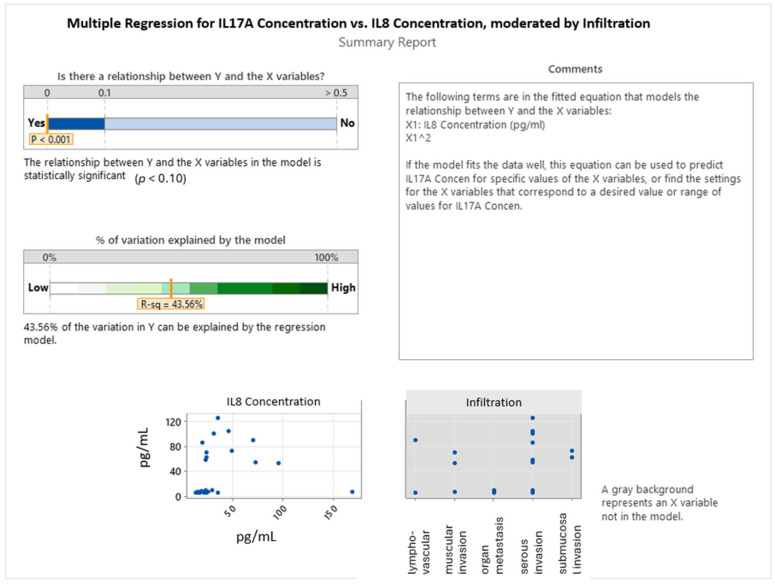
Correlations between IL8 and IL17A in colon cancer invasion.

These findings support a model in which IL-17A expression is modulated by the interplay of IL-8 and IL-33 signaling, with this interaction being further influenced by the tumor’s histopathological grade. The diminished IL-17A response in higher-grade tumors may reflect immune suppression or altered cytokine signaling in more advanced disease states. Collectively, the results underscore the importance of considering interleukin interactions and tumor differentiation jointly when evaluating immune activity in colorectal cancer. The multiple regression model presented in Figure 6 evaluates the influence of IL-8 concentration and tumor infiltration type on IL-17A expression in colorectal cancer. The model is statistically significant (*p* < 0.001), indicating a meaningful relationship between the predictors and IL-17A levels. With an R-squared value of 43.56%, the model explains a moderate proportion of the variation in the IL-17A concentration, suggesting that IL-8 and infiltration collectively account for nearly half of the variability observed. Notably, the model includes a non-linear (quadratic) term for IL-8 concentration, highlighting a curvilinear relationship between IL-8 and IL-17A. This implies that IL-17A levels do not increase linearly with IL-8; rather, IL-17A may rise to a threshold with increasing IL-8 and subsequently decline—suggesting a biphasic or saturation-type response. This dynamic could reflect a regulatory mechanism in the tumor microenvironment, where IL-17A is upregulated under moderate IL-8 levels but potentially suppressed at very high IL-8 concentrations, possibly due to feedback inhibition or immune exhaustion. The inclusion of infiltration type as a variable further refined the model, although its individual contribution was not quantified in this summary. The scatterplots showed variability in IL-17A expression across different patterns of tumor invasion, suggesting that specific invasive behaviors may modulate inflammatory cytokine signaling. In summary, this analysis reveals a significant, non-linear correlation between IL-8 and IL-17A in colorectal cancer, modulated by the type of tumor infiltration. These findings underscore the complexity of cytokine interactions within the tumor microenvironment and highlight the potential of IL-8 and IL-17A as biomarkers reflective of invasion-related immune dynamics.

An ANOVA was performed to assess the variation in the mean IL-17A concentration based on the type of tumor infiltration (lymphovascular invasion, muscular invasion, organ metastasis, serous invasion, submucosal invasion). The analysis revealed no statistically significant differences in mean IL-17A concentrations across the different infiltration types (F(4, 37) = 1.81, *p* = 0.148). Descriptively, the highest mean IL-17A concentration was observed in submucosal invasion (67.88 pg/mL), followed by serous invasion (34.30 pg/mL), muscular invasion (25.1 pg/mL), and lymphovascular invasion (13.41 pg/mL), with the lowest mean in organ metastasis (7.297 pg/mL). While numerical differences were present, the lack of statistical significance at the alpha = 0.05 level suggests that infiltration type alone does not significantly explain the variation in IL-17A levels in this cohort. The model explained 16.35% of the variance in the IL-17A concentration.

## 3. Discussion

Biomarkers in colorectal cancer are essential for early diagnosis, prognosis assessment, treatment selection, and monitoring disease progression, enabling more personalized and effective clinical management [29]. This study investigated the expression levels of interleukins 8, 17A, and 33 in colon cancer, with a focus on tumor grade and patterns of invasion. It also examined epidemiological characteristics, including age and gender distribution, in relation to interleukin expression and disease progression. Furthermore, the study explored correlations among these three interleukins based on tumor aggressiveness [30].

The average age of colon cancer patients appears to vary with tumor grade, and previous studies have reported notable differences across age groups. Younger individuals, particularly those under 40, tend to present with higher-grade tumors characterized by more aggressive histopathological features, including mucinous adenocarcinomas and poorly differentiated subtypes [31]. In contrast, the present study found that the mean age of patients was relatively consistent across different grades of tumor differentiation, with moderately differentiated tumors (G2) being the most prevalent across all age groups. While some studies have linked younger age to a poorer prognosis due to delayed diagnosis and more advanced disease at presentation [32], other investigations have reported no significant differences in TNM staging or histological patterns across age groups [33]. These findings collectively suggest that age may influence tumor grade, anatomical location, and biological behavior—factors critical for guiding therapeutic decision-making. Moreover, our analysis revealed sex-specific trends: in women, poorly differentiated tumors (G3) were more frequently observed at older ages, whereas in men, G2 tumors tended to occur at later ages compared to their female counterparts. The average age of patients with colon cancer is a relevant variable in understanding the extent of tumor invasion. Research suggests that younger individuals, particularly those under 54 years of age, are more likely to present with advanced tumor infiltration, notably at stages T3–T4, although such differences are not always statistically significant [34]. Furthermore, younger patients exhibit a higher incidence of angiolymphatic and perineural invasion, both associated with a poorer prognosis [35]. In contrast, older patients—especially those over 75 years—tend to have lower rates of liver metastases and vascular invasion, which may contribute to improved postoperative outcomes and survival [36]. These observations highlight the influence of age on the invasive behavior of colon cancer and its implications for clinical management. In our cohort, the highest mean age (77 years) was observed in patients with organ metastases, while similar average ages were found in cases of lymphovascular and submucosal invasion. Notably, muscle invasion was associated with the youngest patient group. Our findings suggest that although older age may be associated with more advanced metastatic disease due to delayed diagnosis, younger patients are often diagnosed at earlier stages of local invasion.

The degree of tumor differentiation in colon cancer appears to vary by gender, with several studies reporting notable differences between male and female patients. Women are often diagnosed at an older age than men and have a higher likelihood of developing high-grade tumors in the proximal and distal colon, particularly after menopause [37]. In contrast, men show a greater incidence of colorectal cancer localized to the left colon and rectum, although this distribution does not appear to be associated with significant differences in histological tumor grade [38]. Genetic analyses further support sex-specific patterns, with women more frequently exhibiting mutations in genes, such as BRAF and TGF-β, which are associated with increased tumor aggressiveness, whereas men more commonly display N-RAS mutations, potentially contributing to more rapid disease progression [39]. These findings underscore the importance of gender as a biological variable influencing both histopathological and molecular features of colorectal cancer, with potential implications for diagnostic and therapeutic strategies. In our study, female patients with well- and moderately differentiated tumors (G1 and G2) were, on average, younger than their male counterparts, while poorly differentiated tumors (G3) were more prevalent in older women than in men. Conversely, moderately differentiated tumors were more frequently observed in older male patients. These observations suggest potential gender-related differences in disease progression, with women more susceptible to G3 tumors at advanced ages and men more likely to develop G2 tumors later in life. Gender-based differences in tumor invasion patterns in colon cancer are clinically relevant for prognostic assessment and the development of tailored treatment strategies. Research indicates that women exhibit a higher incidence of right-sided colorectal cancer, a subtype often associated with more aggressive behavior and deeper levels of tumor invasion [40]. This distribution is also linked to a greater frequency of BRAF mutations and high microsatellite instability (MSI-high) in female patients, both of which are correlated with increased tumor invasiveness [39]. In contrast, men more frequently develop tumors in the left colon and rectum, where invasion is commonly associated with N-RAS mutations, potentially contributing to distinct biological behavior and progression patterns [41]. These findings highlight the critical role of gender in shaping the molecular and anatomical characteristics of tumor invasion in colorectal cancer, supporting the need for sex-specific therapeutic considerations. In our study, male patients were generally older than females across most invasion types, particularly in cases of lymphovascular infiltration. However, for organ metastases, the average age was higher among women. These data suggest that advanced tumor invasion in women tends to occur at older ages, whereas in less invasive forms, older age is more commonly observed in men. Such patterns further underscore the influence of gender on disease progression and invasion dynamics in colorectal cancer.

The expression patterns of interleukins IL-8, IL-17A, and IL-33 in colorectal cancer vary according to tumor grade, playing a significant role in disease progression and prognosis. IL-8 is known to promote angiogenesis and tumor advancement, with elevated levels commonly observed in high-grade tumors, where it contributes to increased aggressiveness and metastatic potential [42]. IL-17A is also associated with more aggressive forms of colorectal cancer due to its pro-inflammatory activity and its involvement in tumor progression, particularly in the context of K-RAS mutations [15]. Furthermore, a reduction in IL-17 receptor A (IL-17RA) expression has been reported in advanced tumors, suggesting a diminished cellular response to immune signaling and potential immune evasion [43]. In the case of IL-33, its expression tends to be elevated during the early stages of tumor development but decreases in more advanced and poorly differentiated tumors, indicating a possible dual role—initially protective, followed by a shift toward tumor promotion in later stages [44]. The present study corroborates these findings, showing distinct interleukin expression patterns across tumor grades. IL-8 exhibited the highest mean concentration in well-differentiated tumors (G1), although considerable variability was observed in moderately differentiated tumors (G2), potentially reflecting a heightened and heterogeneous inflammatory response during intermediate stages. In contrast, IL-17A and IL-33 levels decreased progressively with declining tumor differentiation, which may indicate their involvement in early stage inflammatory signaling and the subsequent suppression of immune responses in more advanced cancer. Collectively, these results support the potential utility of IL-8, IL-17A, and IL-33 as biomarkers for assessing tumor behavior and as possible targets for personalized therapeutic interventions in colorectal cancer.

The expression of interleukins IL-8, IL-17A, and IL-33 in colorectal cancer demonstrates a strong association with the depth of tumor invasion across histological layers. IL-8, a critical mediator of angiogenesis and tumor progression, is found at elevated levels in deeply invasive tumors and has been linked to enhanced metastatic potential [45]. IL-17A plays a pivotal role in promoting the epithelial–mesenchymal transition, facilitating cellular migration and invasion, particularly in advanced stages of colorectal cancer. Its expression has been correlated with increased depth of invasion and lymph node metastasis [46]. Similarly, IL-33 has been implicated in tumor metastasis; elevated IL-33 levels are observed in invasive tumors where it contributes to the remodeling of the tumor microenvironment and promotes angiogenesis, thereby supporting disease progression [47]. The present study further reinforces these findings by demonstrating distinct interleukin expression profiles based on the type of tumor invasion. IL-8 concentrations were highest in cases of submucosal and serous invasion, suggesting an intensified inflammatory response in these stages. IL-17A exhibited peak levels in submucosal invasion, followed by a notable decrease in cases with organ metastases, which may reflect diminished immune activation in advanced disease. IL-33 levels were predominantly elevated in muscular and serous invasion, indicating a possible role in facilitating deeper tissue infiltration. Collectively, these results emphasize the relevance of IL-8, IL-17A, and IL-33 in the mechanisms of tumor invasion and support their potential utility as biomarkers and therapeutic targets in colorectal cancer management.

The expression of interleukins IL-8, IL-17A, and IL-33 in colorectal cancer is significantly associated with the degree of tumor differentiation, and their interplay appears to influence tumor progression. While existing literature often suggests that IL-8 overexpression in poorly differentiated tumors contributes to angiogenesis and metastasis [48], our ANOVA did not reveal a statistically significant difference in mean IL-8 concentrations across tumor grades (G1, G2, G3). However, descriptively, G1 tumors showed the highest mean IL-8, and both G1 and G2 exhibited substantial variability, which may indicate a heterogeneous inflammatory landscape or the presence of specific molecular subtypes within these differentiation grades. In contrast, our findings for IL-17A, showing a marked decline with increasing tumor grade (approaching statistical significance with *p* = 0.095 in ANOVA), align with literature suggesting its more prominent expression in well-differentiated tumors and a potentially favorable prognostic role in early stages [49]. For IL-33, our ANOVA similarly showed no statistically significant difference in mean concentrations across tumor grades, despite numerical trends. This suggests that while IL-33 levels are elevated in well-differentiated tumors and decline with decreasing cellular differentiation in some contexts [50], its overall association with tumor grade may be more complex or require larger cohorts for definitive statistical significance.

Despite the individual non-significant or marginally significant associations, an extended multiple regression model incorporating all three interleukins (IL-8, IL-33) and tumor grade demonstrated a statistically significant and robust relationship with IL-17A levels (*p* < 0.001), explaining 70.66% of its variance. This powerful predictive capability underscores the complex, non-linear interplay among these cytokines and their modulation by tumor histological characteristics. Specifically, the model revealed quadratic relationships, as evidenced by the regression equations and interaction plots. For well-differentiated (G1) and moderately differentiated (G2) tumors, IL-17A expression exhibited an inverted U-shaped response to the IL-8 concentration, initially increasing and then declining at very high IL-8 levels. This dynamic suggests that moderate IL-8 concentrations may promote Th17-related inflammation, while excessive IL-8 could lead to immune suppression or a desensitization of IL-17A responses. In contrast, for poorly differentiated (G3) tumors, IL-17A levels remained consistently lower and showed a steeper, continuous decline with increasing IL-8, indicating an attenuated or dysregulated inflammatory response characteristic of more aggressive cancers. Furthermore, IL-33 also exhibited a curvilinear effect on IL-17A, with IL-17A peaking at moderate IL-33 concentrations. These findings suggest that IL-17A expression is intricately modulated by the combined influence of IL-8 and IL-33 signaling, with this interaction being further shaped by the tumor’s histopathological grade. The diminished IL-17A response in higher-grade tumors may reflect immune suppression or altered cytokine signaling in more advanced disease states, highlighting the interconnected roles of IL-8, IL-17A, and IL-33 in colorectal cancer. This complex interaction emphasizes their potential as targets for stratified therapeutic interventions, with IL-17A emerging as a potentially prominent proinflammatory biomarker in early stage, well-differentiated tumors.

The expression and interaction of interleukins IL-8, IL-17A, and IL-33 in colorectal cancer are closely associated with the degree of tumor invasion and play a pivotal role in disease progression. Our findings of the highest IL-8 concentrations in cases of submucosal and serous invasion align with its established role as a key proangiogenic and pro-metastatic cytokine that is significantly upregulated in tumors infiltrating deeper tissue layers and linked to activation of the plasminogen system and increased incidence of hepatic metastases [45]. For IL-17A, its peak levels in submucosal invasion and notable decrease in cases with organ metastases suggest its contribution to tumor invasiveness by promoting the epithelial–mesenchymal transition (EMT) in earlier stages [46], with a potential diminished immune activation in advanced metastatic disease. IL-33 concentrations were predominantly elevated in muscular and serous invasion, with the lowest mean levels in organ metastasis, indicating its possible role in facilitating deeper tissue infiltration. While the role of IL-33 in metastasis remains subject to debate, it has been implicated in creating an immunosuppressive microenvironment and upregulating matrix metalloproteinases MMP-2 and MMP-9 through ST2 receptor activation, which are involved in extracellular matrix degradation [51]. Additionally, IL-33 may indirectly influence IL-17A activity by modulating regulatory T cells (Tregs), thereby contributing to a suppressive tumor immune landscape [50]. These findings collectively point to the complex interplay among IL-8, IL-17A, and IL-33, with each cytokine participating in distinct yet interconnected mechanisms underlying tumor invasion and metastasis. Our multiple regression analysis further revealed a significant, nonlinear correlation between IL-8 and IL-17A in relation to tumor invasion, with the model accounting for 43.56% of the variance in IL-17A expression. This non-linear relationship, where IL-17A levels initially increased with IL-8 concentration but declined beyond a certain threshold, suggests a biphasic inflammatory regulatory mechanism that may reflect immune activation, followed by immune exhaustion or suppression in advanced stages of invasion. In contrast, no significant direct associations were identified between IL-8 and IL-33 or between IL-33 and IL-17A in these regression models, suggesting that IL-33 expression may be regulated by other molecular or microenvironmental factors independent of IL-8 and IL-17A dynamics in this context. Collectively, these findings emphasize the dominant regulatory role of IL-8 in modulating IL-17A activity during tumor invasion and highlight the absence of a direct relationship involving IL-33 in this context. The non-linear correlation between IL-8 and IL-17A further reflects the complexity of cytokine-mediated immune responses in colorectal cancer progression and supports the potential of IL-8 and IL-17A as biomarkers or therapeutic targets based on tumor invasion characteristics.

## 4. Material and Methods

### 4.1. Study Population

This study enrolled patients diagnosed with colon cancer who were scheduled for surgical intervention, alongside a control group comprising 40 healthy individuals (25 females—mean age 66.64, SD 12.31 years, 15 males—mean age 62, SD 10.87 years) undergoing routine colonoscopic evaluation. Cancer patients were recruited over a 12-month period from a specialized medical center. The inclusion criteria covered a range of tumor differentiation grades (G1, G2, G3) and patterns of tumor invasion, including submucosal, muscular, serosal, lymphatic, and distant organ involvement. Participants were stratified according to tumor grade and depth of invasion. Of the 42 patients with colorectal cancer, 8 had grade 1 tumors, 29 had grade 2, and 5 had grade 3. Tumor invasion was classified as submucosal in 2 patients, muscular in 6, serosal in 18, lymph node involvement in 12, and organ metastases in 4. The study cohort included 23 women and 19 men, ranging in age from 45 to 86 years. All participants were fully informed about the objectives and procedures of the study prior to enrollment, and written informed consent was obtained following a comprehensive medical evaluation. Inclusion criteria for the cancer group required a confirmed diagnosis of colorectal adenocarcinoma with histopathological verification of disease stage, no prior exposure to chemotherapy or radiotherapy, and an adequate performance status. Exclusion criteria included the presence of inflammatory conditions that could affect serum cytokine levels, ongoing or recent treatments known to modulate immune function (e.g., chemotherapy, corticosteroids), active infections or other malignancies, recent cardiovascular events (within the last six months), and significant organ dysfunction or injury.

### 4.2. Blood Sample Collection

Venous blood samples were drawn from the median cubital vein of each participant using 2 mL vacutainer glass tubes designed for coagulation. Two tubes were collected per subject. After allowing the blood to clot, samples were centrifuged at 4200 rpm for 10 min at 4 °C. The resulting serum was transferred into sterile Eppendorf tubes and stored at −80 °C until further analysis.

### 4.3. ELISA Analysis

An enzyme-linked immunosorbent assay (ELISA) was employed to quantify the serum levels of IL-33, IL-8, and IL-17A using commercially available kits from BioLegend (San Diego, CA, USA). The specific kits utilized in this study were the LEGEND MAX™ Human IL-33 ELISA Kit (cat. no. 435907), the LEGEND MAX™ Human IL-8 ELISA Kit (cat. no. 431507), and the LEGEND MAX™ Human IL-17A ELISA Kit (cat. no. 433917). These kits are based on the sandwich ELISA principle and are supplied with 96-well strip plates pre-coated with a capture antibody, allowing for sensitive and specific quantification of target proteins in human serum. All assays were conducted in accordance with the manufacturer’s instructions, with each sample analyzed in duplicate wells. Cytokine concentrations were calculated by referencing standard curves generated during each assay. To minimize cross-reactivity and ensure assay precision, each interleukin was measured separately rather than through multiplex analysis. Absorbance measurements were performed using the BioTek Synergy H1 Hybrid microplate reader (Agilent Technologies, Santa Clara, CA, USA).

### 4.4. Statistical Analysis

Statistical analysis of the collected data was performed using Minitab 17 statistical software (Minitab LLC, State College, PA, USA). A comprehensive approach was utilized, incorporating both descriptive and inferential statistical methods to characterize interleukin concentrations and their relationships with clinicopathological features in colorectal carcinoma.

Descriptive statistics were calculated to summarize the central tendency and dispersion of serum interleukin levels (IL-8, IL-17A, and IL-33) across different tumor grades (G1, G2, G3) and invasion types. These included means, standard deviations, and interquartile ranges, providing an overview of the data distribution and variability.

To investigate the predictive relationships between interleukin concentrations, tumor grade, and type of tumor invasion, multiple regression analyses were conducted. These analyses aimed to identify significant associations and quantify the proportion of variance explained by the independent variables. Interaction terms were incorporated into the regression models to explore complex, non-linear relationships and how these associations might be influenced by tumor histological characteristics and invasion patterns.

The statistical significance for all tests was set at a *p*-value < 0.10. This threshold was chosen to identify potential associations in an exploratory context, acknowledging that a *p*-value < 0.05 is the conventional standard for stronger statistical significance. In exploratory studies, particularly those involving biomarker discovery or pilot data with relatively small sample sizes, a slightly relaxed significance level like *p* < 0.10 can be acceptable to minimize Type II errors (false negatives) and to capture potentially relevant biological trends that might not reach the stricter *p* < 0.05 threshold [52,53,54]. This approach prioritizes sensitivity over specificity in the initial identification of potential effects, which can then be further investigated and validated in larger, confirmatory studies [55,56,57].

## 5. Conclusions

This study demonstrated that the expression patterns of interleukins IL-8, IL-17A, and IL-33 are significantly associated with the histological grade and depth of tumor invasion in colorectal cancer, offering valuable insights into tumor aggressiveness and immune dynamics. IL-8 levels were highest in well-differentiated tumors and in cases of submucosal and serosal invasion, suggesting its early involvement in inflammatory signaling and its role in promoting angiogenesis and metastatic potential. IL-17A showed peak expression in submucosal invasion but declined with tumor dedifferentiation and in metastatic stages, indicating a proinflammatory role in early tumor progression that diminishes as immune suppression intensifies. IL-33 expression was elevated in muscular and serosal invasion but decreased in poorly differentiated and metastatic tumors, suggesting a dual role—potentially protective in early stages and tumor-promoting in advanced disease. Multiple regression analysis revealed a nonlinear, statistically significant correlation between IL-8 and IL-17A, where IL-17A initially rises with increasing IL-8 but declines beyond a critical threshold, possibly reflecting a transition from immune activation to exhaustion. No significant associations were found between IL-33 and the other interleukins, suggesting distinct regulatory mechanisms. Importantly, a multivariate model including IL-8, IL-17A, IL-33, and tumor grade accounted for over 70% of the variance in IL-17A expression, reinforcing the complex interplay among these cytokines in shaping the tumor microenvironment. These findings support the potential of IL-8 and IL-17A as dynamic biomarkers of tumor behavior and underscore the importance of interleukin profiling in guiding personalized therapeutic strategies in colorectal cancer.

## 6. Study Limitations

While the findings of this study offer valuable insights into the relationship between interleukin expression and colorectal cancer progression, several limitations should be acknowledged. The relatively small sample size and uneven distribution across tumor grades and invasion types may limit the statistical robustness and generalizability of the results. Additionally, as a single-center, cross-sectional analysis, the study does not capture longitudinal changes in cytokine expression or allow for causal inferences. Serum-based measurements, while informative, may not fully reflect local interleukin activity within the tumor microenvironment. Moreover, although key exclusion criteria were applied, the influence of other potential confounding factors could not be entirely ruled out. Lastly, the absence of functional assays and clinical outcome data limits the ability to assess the prognostic or therapeutic implications of the findings. These considerations highlight opportunities for further research in larger, multi-center cohorts with complementary molecular and clinical analyses. The absence of functional assays and clinical outcome data limits the ability to assess the prognostic or therapeutic implications of the findings. These considerations highlight opportunities for further research in larger, multi-center cohorts with complementary molecular and clinical analyses.

## Figures and Tables

**Table 1:** Age and gender distribution in colon cancer grading.

Gender	Degree of Differentiation	N	N*	Mean Age	SE Mean	StDev	Minimum	Q1	Median	Q3	Maximum
F	G1	4	0	65.00	6.57	13.14	47.00	51.50	67.50	76.00	78.00
F	G2	15	0	67.60	3.26	12.64	48.00	56.00	69.00	78.00	85.00
F	G3	4	0	70.75	5.68	11.35	59.00	60.25	70.00	82.00	84.00
M	G1	4	0	68.00	3.19	6.38	62.00	63.00	66.50	74.50	77.00
M	G2	14	0	70.93	03.08	11.52	45.00	63.00	71.50	81.25	86.00
M	G3	1	0	62.00	*	*	62.00	*	62.00	*	62.00

N: Number of observations/patients in the group. N*: Number of missing values or cases excluded due to specific criteria (e.g., single observation where standard deviation cannot be calculated, as seen in male G3 in Table 1). SE Mean: Standard Error of the Mean. StDev: Standard Deviation. Q1: First Quartile. Q3: Third Quartile. *: there are no values in that interval.

**Table 2:** Age and gender distribution in colon cancer invasion.

Invasion Type	Variable	Gender	N	N*	Mean	SE Mean	StDev	Minimum	Q1	Median	Q3	Maximum
Lymphovascular Invasion	Age	F	7	0	62.14	3.87	10.24	48.00	51.00	64.00	73.00	75.00
	Age	M	5	0	71.60	4.70	10.50	59.00	62.50	68.00	82.50	83.00
Muscular Invasion	Age	F	5	0	64.00	4.38	9.80	47.00	56.00	67.00	70.50	71.00
	Age	M	1	0	67.00	*	*	67.00	*	67.00	*	67.00
Organ Metastasis	Age	F	2	0	80.00	4.00	5.66	76.00	*	80.00	*	84.00
	Age	M	2	0	74.00	12.00	17.00	62.00	*	74.00	*	86.00
Serous Invasion	Age	F	8	0	73.25	4.61	13.05	51.00	60.00	78.00	84.00	85.00
	Age	M	10	0	67.60	3.39	10.72	45.00	61.50	67.50	77.25	81.00
Submucosal Invasion	Age	F	1	0	56.00	*	*	56.00	*	56.00	*	56.00
	Age	M	1	0	78.00	*	*	78.00	*	78.00	*	78.00

N: Number of observations/patients in the group. N*: Number of missing values or cases excluded due to specific criteria (e.g., single observation where standard deviation cannot be calculated, as seen in male G3 in Table 1). SE Mean: Standard Error of the Mean. StDev: Standard Deviation. Q1: First Quartile. Q3: Third Quartile. *: there are no values in that interval.

**Table 3:** Interleukin concentration in colon cancer grading.

Variable	Degree of Differentiation	N	N*	Mean	SE Mean	StDev	Minimum	Q1	Median	Q3	Maximum
IL8 Concentration (pg/mL)	G1	8	0	39.4	10.5	29.8	13.8	18.6	24.4	66.2	95.3
	G2	29	0	26.75	5.54	29.85	13.78	15.50	16.76	23.90	168.56
	G3	5	0	26.73	3.11	6.95	17.22	20.78	26.18	32.95	36.05
IL17A Concentration (pg/mL)	G1	8	0	47.4	13.4	37.8	6.3	6.7	54.4	79.5	104.8
	G2	29	0	23.40	6.43	34.64	5.80	6.34	6.53	9.16	126.00
	G3	5	0	7.306	0.702	1.569	6.254	6.390	6.617	8.566	10.062
IL33 Concentration (pg/mL)	G1	8	0	48.1	20.5	57.9	6.1	14.6	31.6	50.1	186.5
	G2	29	0	43.53	7.26	39.10	8.07	20.32	33.36	44.61	171.90
	G3	5	0	27.3	13.2	29.6	6.6	7.4	10.9	55.4	76.1

N: Number of observations/patients in the group. N*: Number of missing values or cases excluded due to specific criteria (e.g., single observation where standard deviation cannot be calculated, as seen in male G3 in Table 1). SE Mean: Standard Error of the Mean. StDev: Standard Deviation. Q1: First Quartile. Q3: Third Quartile.

**Table 4:** Interleukin concentration in colon cancer invasion.

Variable	Infiltration	N	N*	Mean	SE Mean	StDev	Minimum	Q1	Median	Q3	Maximum
IL8 Concentration (pg/mL)	lymphovascular invasion	12	0	22.57	4.52	15.66	15.39	15.62	16.53	23.65	70.85
	muscular invasion	6	0	32.2	12.8	31.3	13.8	15.7	21.3	42.6	95.3
	organ metastasis	4	0	24.51	5.06	10.11	14.93	15.50	23.54	34.50	36.05
	serous invasion	18	0	32.73	8.71	36.95	13.78	15.44	19.60	32.69	168.56
	submucosal invasion	2	0	36.7	12.4	17.5	24.4	*	36.7	*	49.1
IL17A Concentration (pg/mL)	lymphovascular invasion	12	0	13.41	7.02	24.32	5.80	6.28	6.44	6.59	90.64
	muscular invasion	6	0	25.1	11.9	29.2	6.3	6.4	6.9	58.1	70.5
	organ metastasis	4	0	7.297	0.924	1.847	6.254	6.277	6.436	9.178	10.062
	serous invasion	18	0	34.30	9.96	42.25	5.80	6.50	7.61	65.87	126.00
	submucosal invasion	2	0	67.88	5.73	8.11	62.14	*	67.88	*	73.61
IL33 Concentration (pg/mL)	lymphovascular invasion	12	0	35.85	7.69	26.62	9.00	12.74	28.28	59.64	91.18
	muscular invasion	6	0	49.1	27.9	68.3	6.1	9.2	28.8	72.5	186.5
	organ metastasis	4	0	32.6	16.4	32.7	6.6	7.0	23.8	67.0	76.1
	serous invasion	18	0	47.2	10.7	45.5	8.1	22.0	31.9	43.5	171.9
	submucosal invasion	2	0	39.26	4.71	6.66	34.55	*	39.26	*	43.97

N: Number of observations/patients in the group. N*: Number of missing values or cases excluded due to specific criteria (e.g., single observation where standard deviation cannot be calculated, as seen in male G3 in Table 1). SE Mean: Standard Error of the Mean. StDev: Standard Deviation. Q1: First Quartile. Q3: Third Quartile. *: there are no values in that interval.

## Data Availability

The raw data supporting the conclusions of this article will be made available by the authors on request.

## References

[1] Thuraisingam R., Jandova J., Pandit V., Michailidou M., Nfonsam V.N. (2017). Assessing the National Trends in Colon Cancer among Native Americans: A 12 Year SEER Database Study. Am. J. Surg..

[2] McCarthy M. (2014). US Colon Cancer Rate Dropped 30% among over 50s in Past Decade. BMJ.

[3] Shah N., Acuna-Villaorduna A., Goel S. (2019). Incidence Patterns of Early Onset Colon Cancer by Race and Stage in the US. J. Clin. Oncol..

[4] Saika K., Machii R. (2016). Subsite Distribution of Colon Cancer from Cancer Incidence in Five Continents Vol. X. Jpn. J. Clin. Oncol..

[5] Mitry E., Rachet B., Quinn M.J., Cooper N., Coleman M.P. (2008). Survival from Cancer of the Colon in England and Wales up to 2001. Br. J. Cancer.

[6] Chopra M., Singh K., Verma N. (2012). Autoimmune and Inflammatory Diseases Biomarkers. Int. J. Biomed. Adv. Res..

[7] Medina-Leyte D.J., Zepeda-García O., Domínguez-Pérez M., González-Garrido A., Villarreal-Molina T., Jacobo-Albavera L. (2021). Endothelial Dysfunction, Inflammation and Coronary Artery Disease: Potential Biomarkers and Promising Therapeutical Approaches. Int. J. Mol. Sci..

[8] Normanno N., Apostolidis K., de Lorenzo F., Beer P.A., Henderson R., Sullivan R., Biankin A.V., Horgan D., Lawler M. (2022). Cancer Biomarkers in the Era of Precision Oncology: Addressing the Needs of Patients and Health Systems. Semin. Cancer Biol..

[9] Farc O., Budisan L., Zaharie F., Țăulean R., Vălean D., Talvan E., Neagoe I.B., Zănoagă O., Braicu C., Cristea V. (2024). Expression and Functional Analysis of Immuno-Micro-RNAs Mir-146a and Mir-326 in Colorectal Cancer. Curr. Issues Mol. Biol..

[10] Tâlvan E.-T., Mohor C.I., Chisnoiu D., Făgețan I.M., Tâlvan C.-D., Cristea V., Câmpian R.S. (2018). Correlation of Chronic Periodontitis Progression with sTREM-1 and E-Cadherin Salivary Levels. Rev. Romana Med. Lab..

[11] Tâlvan C.-D., Budișan L., Tâlvan E.-T., Grecu V., Zănoagă O., Mihalache C., Cristea V., Berindan-Neagoe I., Mohor C.I. (2024). Serum Interleukins 8, 17, and 33 as Potential Biomarkers of Colon Cancer. Cancers.

[12] Gulubova M., Chonov D., Aleksandrova E., Ivanova K., Ignatova M.M., Vlaykova T. (2024). Interleukin-6-Positive Immune Cells as a Possible New Immunologic Marker Associated with the Colorectal Cancer Prognosis. Appl. Immunohistochem. Mol. Morphol. AIMM.

[13] Smagina E., Polit’ko D., Kumeiko V., Gurina L., Stenkova A. (2025). Correlation Between Interleukin IL-6/IL-6 Receptor Polymorphisms (IL6–174C>G and IL6R 1073A>C) and RAS/BRAF Mutations in Patients with Colorectal Cancer. Gastroenterol. Insights.

[14] Banday M.Z., Sameer A.S., Chowdri N.A., Haq E. (2017). Interleukin-10 -592C/A, but Not -1082A/G Promoter Single Nucleotide Polymorphism, Is Associated with a Decreased Risk of Colorectal Cancer in an Ethnic Kashmiri Population: A Case-Control Study. Eur. J. Cancer Prev..

[15] Omrane I., Marrakchi R., Baroudi O., Mezlini A., Ayari H., Medimegh I., Stambouli N., Kourda N., Bouzaienne H., Uhrhammer N. (2014). Significant Association between Interleukin-17A Polymorphism and Colorectal Cancer. Tumour Biol. J. Int. Soc. Oncodev. Biol. Med..

[16] Najdaghi S., Razi S., Rezaei N. (2020). An Overview of the Role of Interleukin-8 in Colorectal Cancer. Cytokine.

[17] Sun Q., Sun F., Wang B., Liu S., Niu W., Liu E., Peng C., Wang J., Gao H., Liang B. (2014). Interleukin-8 Promotes Cell Migration through Integrin Avβ6 Upregulation in Colorectal Cancer. Cancer Lett..

[18] Conciatori F., Bazzichetto C., Falcone I., Ferretti G., Cognetti F., Milella M., Ciuffreda L. (2019). Colorectal Cancer Stem Cells Properties and Features: Evidence of Interleukin-8 Involvement. Cancer Drug Resist..

[19] Xia W., Chen W., Zhang Z., Wu D., Wu P., Chen Z., Li C., Huang J. (2015). Prognostic Value, Clinicopathologic Features and Diagnostic Accuracy of Interleukin-8 in Colorectal Cancer: A Meta-Analysis. PLoS ONE.

[20] Yang C.-Y., Lin C.-H., Jiang J.-K. (2017). The Expression of Interleukin 17 Receptor A Is Associated with Poor Prognosis in Patients with Colorectal Cancer and Its Knockdown Inhibits Tumor Growth and Modulates Tumor-Infiltrating Immune Cells in Mice Tumor. Ann. Oncol..

[21] Jiang J.-K., Lin C.-H., Chang T.-A., Lo L.-C., Lin C.-P., Lu R.-H., Yang C.-Y. (2024). Decreased Interleukin-17RA Expression Is Associated with Good Prognosis in Patients with Colorectal Cancer and Inhibits Tumor Growth and Vascularity in Mice. Cancer Med..

[22] Li S., Na R., Li X., Zhang Y., Zheng T. (2022). Targeting Interleukin-17 Enhances Tumor Response to Immune Checkpoint Inhibitors in Colorectal Cancer. Biochim. Biophys. Acta Rev. Cancer.

[23] Sui G., Qiu Y., Yu H., Kong Q., Zhen B. (2019). Interleukin-17 Promotes the Development of Cisplatin Resistance in Colorectal Cancer. Oncol. Lett..

[24] Yang W., Chen J., Liang H., Wu W. (2022). Interleukin-17A mRNA Expression Is Associated with the Prognosis of Patients with Colorectal Cancer: A Pooled Meta-Analysis. Turk. J. Gastroenterol..

[25] Zhou Y., Ji Y., Wang H., Zhang H., Zhou H. (2018). IL-33 Promotes the Development of Colorectal Cancer Through Inducing Tumor-Infiltrating ST2L+ Regulatory T Cells in Mice. Technol. Cancer Res. Treat..

[26] Cui G., Yuan A., Pang Z., Zheng W., Li Z., Goll R. (2018). Contribution of IL-33 to the Pathogenesis of Colorectal Cancer. Front. Oncol..

[27] Wang A., Gong Z., Meng J., Liu H., Gao J. (2019). Relationship between serum soluble interleukin-33 levels and clinical features in patients with colorectal cancer and its diagnostic value. Acta medica mediterranea..

[28] Eissmann M.F., Dijkstra C., Wouters M.A., Baloyan D., Mouradov D., Nguyen P.M., Davalos-Salas M., Putoczki T.L., Sieber O.M., Mariadason J.M. (2018). Interleukin 33 Signaling Restrains Sporadic Colon Cancer in an Interferon-γ-Dependent Manner. Cancer Immunol. Res..

[29] Tâlvan C.-D., Tâlvan E.-T., Mohor C.I., Budișan L., Grecu V., Mihalache M., Zănoagă O., Chira S., Berindan-Neagoe I., Cristea V. (2024). Exploring miRNA Profiles in Colon Cancer: A Focus on miR101-3p, miR106a-5p, and miR326. Cancers.

[30] Tâlvan C.-D., Tâlvan E.-T., Mohor C.I., Budișan L., Grecu V., Mihalache M., Neagoe I.B., Zănoagă O., Oprinca G.C., Cristian A.N. (2025). The Impact of miRNA Expression on Colon Cancer Severity, Invasiveness, and Localization. Cancers.

[31] da Fonseca L.M., da Luz M.M.P., Lacerda-Filho A., Cabral M.M.D.A., da Silva R.G. (2012). Colorectal Carcinoma in Different Age Groups: A Histopathological Analysis. Int. J. Color. Dis..

[32] Abdulrazzaq T.A., Salayi J.S. (2023). Clinicopathological Comparison of Colorectal Cancer in Young and Old Patients. AMJ Adv. Med. J..

[33] Bossola M., Sofo L., Ratto C., Bellantone R., Doglietto G.B., Crucitti A., Crucitti F. (1989). Colorectal Cancer in Patients 40 Years of Age and Younger. Ital. J. Surg. Sci..

[34] Mulyati Sri R., Khairunnisa Z., Nabil Ibnu S. (2023). Relationship Between Age and Depth of Invasion and Histopathological Features in Colorectal Cancer Patients in the Anatomical Pathology Department of Cut Meutia General Hospital (RSUCM) 2018 - 2021. J. Multidisiplin. Madani.

[35] Silva F.M.M.D., Duarte R.P., Leão C.C.A., Vissoci C.M., Alvarenga A.L.A.T., Ramos A.B.S., Goulart A.E.C. (2020). Colorectal Cancer in Patients under Age 50: A Five-Year Experience. Rev. Colégio Bras. Cir..

[36] Hessman O., Bergkvist L., Ström S. (1997). Colorectal Cancer in Patients over 75 Years of Age—Determinants of Outcome. Eur. J. Surg. Oncol..

[37] Brozek W., Kriwanek S., Bonner E., Peterlik M., Cross H.S. (2009). Mutual Associations between Malignancy, Age, Gender, and Subsite Incidence of Colorectal Cancer. Anticancer Res..

[38] Ram E., Zbar A., Bachar G., Krissi H., Joubran S., Rath-Wolfson L. (2013). Gender Differences in Colorectal Cancer Presentation and Outcome at a Single Israeli Institution: 1989–2002. Acta Chir. Iugosl..

[39] Tsai Y.-J., Huang S.-C., Lin H.-H., Lin C.-C., Lan Y.-T., Wang H.-S., Yang S.-H., Jiang J.-K., Chen W.-S., Lin T.-C. (2018). Differences in Gene Mutations According to Gender among Patients with Colorectal Cancer. World J. Surg. Oncol..

[40] Kim S.-E., Paik H.Y., Yoon H., Lee J.E., Kim N., Sung M.-K. (2015). Sex- and Gender-Specific Disparities in Colorectal Cancer Risk. World J. Gastroenterol..

[41] Yang Y., Wang G., He J., Ren S., Wu F., Zhang J., Wang F. (2017). Gender Differences in Colorectal Cancer Survival: A Meta-Analysis. Int. J. Cancer.

[42] Bărbălan A., Streața I., Ivan E.T., Cherciu I., Șurlin V., Ioana M., Săftoiu A. (2017). Interleukin-8 mRNA Expression in Locally Advanced Colorectal Cancer Patients. Curr. Health Sci. J..

[43] Chai B.Y., Yip W.K., Dusa N., Mohtarrudin N., Seow H.F. (2020). Loss of Interleukin-17RA Expression Is Associated with Tumour Progression in Colorectal Carcinoma. Pathol. Oncol. Res. POR.

[44] Cui G., Qi H., Gundersen M.D., Yang H., Christiansen I., Sørbye S.W., Goll R., Florholmen J. (2015). Dynamics of the IL-33/ST2 Network in the Progression of Human Colorectal Adenoma to Sporadic Colorectal Cancer. Cancer Immunol. Immunother. CII.

[45] Terada H., Urano T., Konno H. (2005). Association of Interleukin-8 and Plasminogen Activator System in the Progression of Colorectal Cancer. Eur. Surg. Res..

[46] Chen Y., Yang Z., Wu D., Min Z., Quan Y. (2019). Upregulation of interleukin-17F in Colorectal Cancer Promotes Tumor Invasion by Inducing Epithelial-mesenchymal Transition. Oncol. Rep..

[47] Zhang Y., Davis C., Shah S., Hughes D., Ryan J.C., Altomare D., Peña M.M.O. (2017). IL-33 Promotes Growth and Liver Metastasis of Colorectal Cancer in Mice by Remodeling the Tumor Microenvironment and Inducing Angiogenesis. Mol. Carcinog..

[48] Farc O., Berindan-Neagoe I., Zaharie F., Budisan L., Zănoagă O., Cristea V. (2021). Characterization of the Immune Response Through Cytokine Profiling and Correlational Analysis in Colorectal Malignant Tumors. Rom. Arch. Microbiol. Immunol..

[49] Lin Y., Xu J., Su H., Zhong W., Yuan Y., Yu Z., Fang Y., Zhou H., Li C., Huang K. (2015). Interleukin-17 Is a Favorable Prognostic Marker for Colorectal Cancer. Clin. Transl. Oncol..

[50] Pastille E., Wasmer M.-H., Adamczyk A., Vu V.P., Mager L.F., Phuong N.N.T., Palmieri V., Simillion C., Hansen W., Kasper S. (2019). The IL-33/ST2 Pathway Shapes the Regulatory T Cell Phenotype to Promote Intestinal Cancer. Mucosal Immunol..

[51] Liu X., Zhu L., Lu X., Bian H., Wu X., Yang W., Qin Q. (2014). IL-33/ST2 Pathway Contributes to Metastasis of Human Colorectal Cancer. Biochem. Biophys. Res. Commun..

[52] How to Determine the Right Significance Level for Your Test. https://www.statsig.com/perspectives/right-significance-level-test.

[53] Hayat M.J. (2010). Understanding Statistical Significance. Nurs. Res..

[54] Mohr L.B. (1990). Understanding Significance Testing.

[55] Haile Z.T. (2023). Power Analysis and Exploratory Research. J. Hum. Lact..

[56] Riley R.D., Ahmed I., Ensor J., Takwoingi Y., Kirkham A., Morris R.K., Noordzij J.P., Deeks J.J. (2015). Meta-Analysis of Test Accuracy Studies: An Exploratory Method for Investigating the Impact of Missing Thresholds. Syst. Rev..

[57] Petriella K. Significance Levels 101 for Smarter Experimentation. https://www.geteppo.com/blog/significance-levels-for-smarter-experimentation.

